# A novel method of bilateral patent processus vaginalis ligation in transumbilical single-site multiport laparoscopic orchiopexy

**DOI:** 10.7150/ijms.44682

**Published:** 2020-04-15

**Authors:** Xin Wang, Yong Guan, Yong Wu, QingYa Meng, Ming Dong

**Affiliations:** 1 Department of Pediatric Surgery, Tianjin Children's Hospital, No.238 LongYan Road, Tianjin 300134, PR China; 2 Department of lung cancer surgery, Tianjin medical university general hospital, No.154 Anshan Road, Tianjin 300052, PR China.

**Keywords:** laparoscopic surgery, bilateral patent processus vaginalis, cryptorchidism

## Abstract

**Objective:** To evaluate safety and efficacy of a novel method of bilateral patent processus vaginalis ligation in transumbilical single-site multiport laparoscopic orchiopexy for children.

**Methods:** A retrospective study was carried out comparing the novel ligation and conventional ligation performed by a single surgeon between July, 2017-July, 2018. The patients were divided into the novel group (42 cases) and the conventional group (59 cases). In the novel group, transumbilical single-site multiport laparoscopic orchiopexy was performed and the bilateral internal rings was stitched with “8” pattern suture. In the conventional group, the conventional TriPort laparoscopic orchiopexy was performed and purse string suture was used to fix the internal rings. The parameters of operative duration time, postoperative hospital stay; postoperative complications were compared between 2 groups.

**Results:** All operations were successful. No Perioperative period complications were found and all patients were discharged within 4-6 days after operation. There is no statistic difference in the surgery time and hospitalization day. However, there is significant difference in the Pain face scale scores after day 2(1.60±0.73 VS 2.02±0.86). And there is no scar and the satisfactory cosmetic could be seen in scrotum and inguinal area in the novel group.

**Conclusion:** The novel ligation was safety and efficacy. It is relatively easy to perform with smaller scar and less pain. We propose the novel ligation as a more viable treatment option for pediatric cryptorchidism with bilateral patent processus vaginalis.

## Introduction

Cryptorchidism is the most common male genital anomaly identified, affecting about 3% of male neonates and up to 30% of premature infants 1. The one or both testicles aren't only position in the scrotum appropriately but also can't be moved into the proper position manually. The orchiopexy (place and fix the testicles in the scrotum) is the most common surgical treatment method. In case of the testicle is found to be atrophic with little or no viable germ cell tissue remaining, the orchiectomy is performed 2. Traditional open surgery was the most popular surgical intervention in the past 3. But because of the difficulty of surgical mobilize the peeping tests, complication and testicular atrophy (3~18%), laparoscopy has been hypothesized to be an effective method and become an important diagnostic and treatment tool for cryptorchidism 2,4. And a recently study suggests that laparoscopy is a great and safety option for patients with palpable undescended testes, regardless of its position in the inguinal canal 5. The conventional laparoscopic need 3~4 incisions. And to improve the benefits of laparoscopy concerning post-operative pain, rapid recovery and cosmesis, transumbliical laparoendoscopic single-site surgery (LESS) for pediatrics has been performed in urologic surgery 6. However, the application of LESS has been limited because it required skilled laparoscopic techniques and special instruments. Compared with LESS, tranumbilical multi-port laparoscopic orchiopexy has advantage of easier technique and better cosmetic result 7. Cryptorchidism with bilateral processus vaginalis unclosed is very common; however, less information is available about how to manage the bilateral processus vaginalis unclosed by tranumbilical laparoscopic surgery.

Our department performed tranumbilical multi-port laparoscopic orchiopexy for pediatric cryptorchidism with bilateral processus vaginalis unclosed from 2017. And we'd like to introduce our experience and report the results of intervention on cryptorchidism with bilateral processus vaginalis unclosed.

## Patients and Methods

Our current series consists of 101 procedures at our department. Between 2017-July and 2018 July, all children with newly diagnosed cryptorchidism with bilateral processus vaginalis unclosed include the study. Table 1 lists details of subjects.42 patients who underwent the novel ligation of bilateral processus vaginalis and tranumbilical multi-port laparoscopic orchiopexy (novel group) and 59 patients who underwent conventional ligation and TriPort laparoscopic orchiopexy (conventional group). Laparoscopic orchipexy and the internal rings fixation were successively performed on all patients by a single surgeon (Yong, Guan). Patients were followed-up 1month after stent removal and then 3 monthly thereafter for at least 1year. Postoperative outcomes were mainly evaluated by ultrasound. Surgical technique, complications, and clinical outcomes were reviewed. The informed consent was obtained for diagnostic laparoscopy and orchidopexy.

All 101 patients were performed routine preoperative preparation and standard anesthesia protocol. An appropriately 103 sized endotracheal tube was used in mechanically ventilated for all patients. All patients were placed in the supine position.

### Surgical Technique for transumbilical single-site multiport laparoscopic orchiopexy with the novel ligation of bilateral patent processus vaginalis

**Figure 1:**
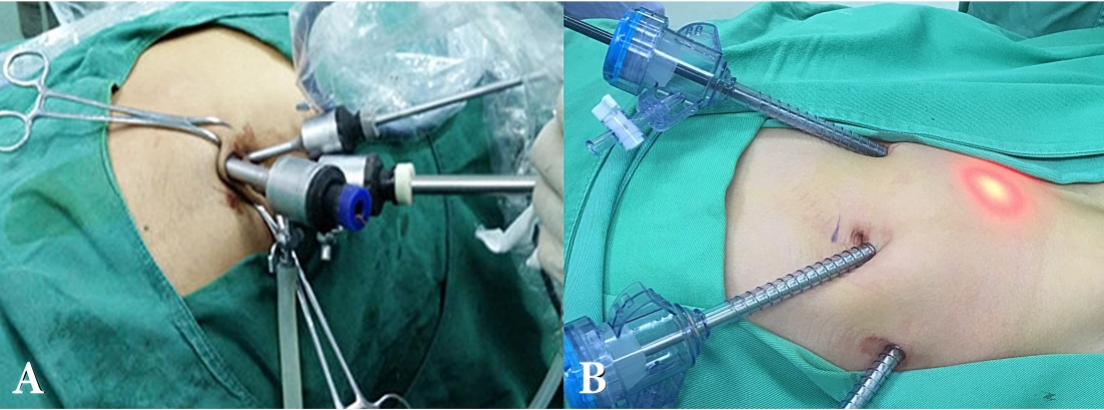
The incision of the surgery.** A)** The incision of LESS group. **B)** The incision conventional laparoscopic group.

**Figure 2:**
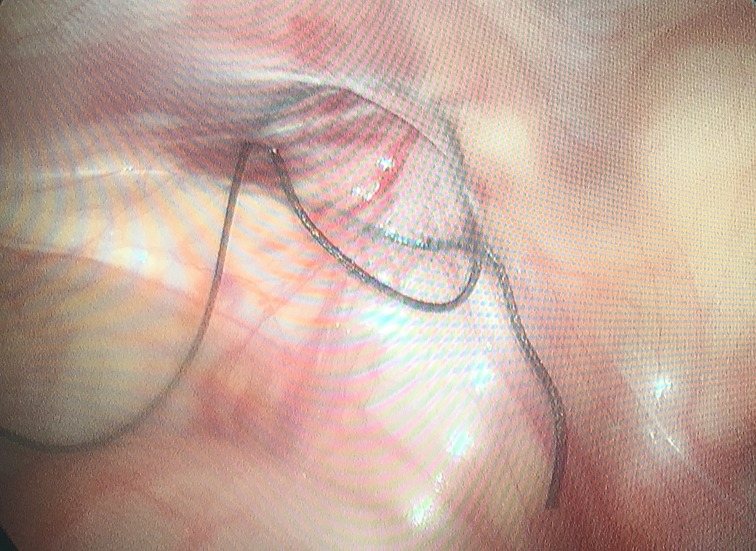
The opposite internal were stitched by 2-0 silk “8” pattern with needle.

We performed a 5-mm umbilical incision for all patients and then inserted a 5-mm trocar. A 5-mm 30-degree lens was introduced and pneumoperitoneum was achieved to 8-12 mmHg. Explored and confirmed spermatic cords and the location and development of affected testes, as well as the contralateral situation. Other two operating ports were made, making a triangle around the umbilicus with the trocar (Fig 1A). The peritoneum was mobilized off of the gonadal vessels. We made a peritoneal incision medial to the vas deferens, divided the gubernaculum and pulled it into the abdomen, extended the proximally to optimize mobilization for a tension free orchidopexy. When testis gubernaculum was clamped and pulled through extra-corporeally, the clamp could further dissect the processus vaginalis off of the spermatic cord for mobility. When adequate testicular mobilization was achieved, the testis was re-located into scrotal through the internal ring. We performed stage Fowler- Stephens orchiopexy. The descended testis was re-located into scrotal pouch using 5-0 non-absorbable suture. The orchiectomy was performed if the teste was necrosis and atrophy. The internal rings of the affected side were stitched with a needle between the edge of arcuate of muscular traverses abdominis and fascia transversalis of posterior peritoneum. And the bilateral internal were stitched by 2-0 silk “8” pattern with needle (Fig 2).

### Surgical technique for conventional TriPort laparoscopic orchiopexy with the traditional ligation of bilateral patent processus vaginalis

A 5.5 mm umbilical trocar was inserted, confirming the bilateral situation of location and development of testes and spermatic cords. Two additional 5.5 mm trocars were inserted in the midclavicular line just on the level of the umbilicus (Fig 1B). The orchiopexy and orchiectomy were performed similar as FEWER groups. However, purse string suture was done for bilateral internal rings of all cases.

### Statistical Analysis

Data analysis was performed using SPSS 21.0. Means (±SD) was used to describe the characteristics. All values were expressed as median values with range. Student's t-test was used to compare the operative time was compared using student's t-test. A value of P < 0.05 denoted statistical significance.

## Results

**Figure 3:**
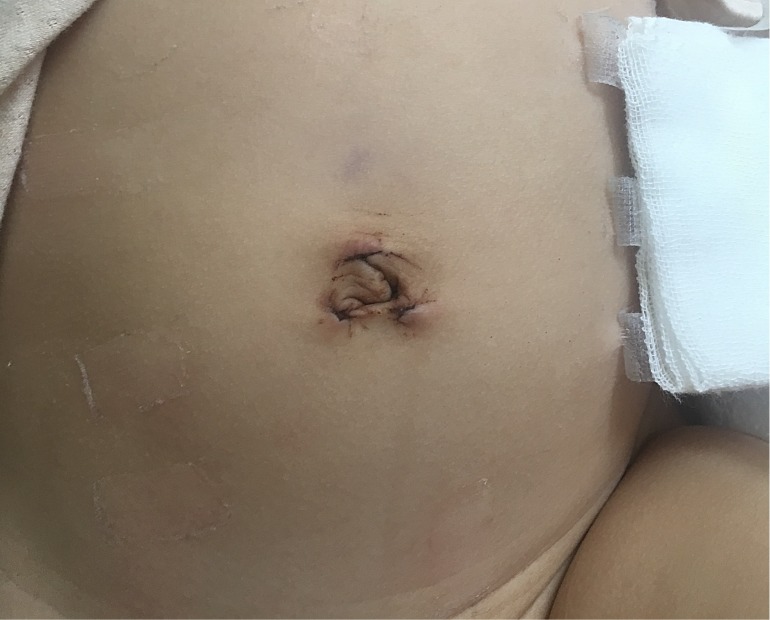
There is no scar and the satisfactory cosmetic could be seen in scrotum and inguinal area in LESS group.

All 101 undescended testes were placed into the scrotum successfully. No Perioperative period complications such as bleeding, abdominal visceral injury, scrotal hematoma and post-operative infection were found in our study and all patients discharged within 4-6 days after operation. There is no statistic difference in the surgery time and hospitalization day. However, there is significant difference in the Pain face scale scores on day 2(1.60±0.73 VS 2.02±0.86) and day 3 (0.81±0.59VS1.98±0.92). There is no scar and the satisfactory cosmesis could be seen in scrotum and inguinal area in novel group (Table 2) (Fig 3). Median follow-up time was 16 months (range, 12-18 months). No patient was lost to follow-up. Normal size and location of testis were indicated by ultrasound examination and telephone inquiring during follow-up periods. We didn't find the inguinal hernia, wound infection, hydrocele, fever, suture granuloma formation, fever, re-ascending, and atrophy in further consultation in out-patient department.

## Discussion

Laparoscopic orchiopexy is a successful, eventless approach for peeping testis, especially for patients with palpable undescended testes, regardless of its position in the inguinal canal 5. Laparoendoscopic single-site surgery (LESS) for pediatrics has been performed in urologic surgery to reduce 3 or 4 incisions to 1 incision and improve the benefits such as postoperative pain, rapid recovery and cosmesis. Despite the cosmetic advantages of LESS, the overall benefits of LESS concerning intraoperative safety and postoperative recovery remain unclear. In addition, in many countries, especially developing countries, the lack of specific instruments, such as multichannel laparoscopic ports, curved instruments, or flexible laparoscopes, limits the wide use of LESS in pediatric patients 8. Moreover, the learning curve to master the technical skills also hampers the performance of true LESS 9. Compared with LESS, tranumbilical multi-port laparoscopic orchiopexy has advantage of easier technique and better cosmetic result 7. But beyond that, another difficult point of LESS is bilateral patent processus vaginalis repair, especially for contralateral repair. Because of the small operation space, interference between different surgical instruments and conventional purse string suture 10. Some study suggest that laparoscopic repair of patent processus vaginalis is feasible using this technique of complete resection without suture and fixation just for the rings less than 10 mm^11^. But there is no report in the literature suggesting the method of ligation and fixation for the rings more than 10mm.

In our study, all rings were wider than 10mm. The circle was sutured with “8” pattern suture instead of conventional purse string suture to reduce the operation time and difficulty. The operative time for tranumbilical multi-port laparoscopic orchiopexy was no longer than conventional laparoscopic repair in this report. Blood loss was minimal, and no intraoperative and postoperative complications were observed in either method. In the present study, there was significant difference in the pain face scales after day 2. In the novel group, the pain subsided more quickly after surgery. All patients made comments about their satisfaction with the cosmetic appearance in the novel group because of the minimal visible scar. To the best of our knowledge, we report the first series utilizing “8” pattern suture for tranumbilical multi-port laparoscopic orchiopexy with bilateral patent processus vaginalis repair in the pediatric population. It suggests the benefits are lower visual scar and less pain without increasing the operation difficulty, operation time and complication. In pediatric patients, surgery scar is expected to expand with growth. No extra-umbilical incisions are required in our method. Therefore, compared with the two or three incisions of 5 to 10 mm in conventional laparoscopic surgery, this method has a significant cosmetic difference.

Tranumbilical multi-port laparoscopic surgery is slightly challenging because operators have less freedom of movement with all instruments using the same entry point. However, the novel “8” pattern suture method to fix the bilateral patent processus vaginalis is not only safety and efficacy but also a simpler method than conventional purse string suture. And our method has technical advantage in decreasing the visible scar and pain.

## Conclusions

The advantages of tranumbilical multi-port laparoscopic surgery superior aesthetics with a smaller scar and less pain. And “8” pattern suture method to fix bilateral patent processus vaginalis is not only safety and efficacy but also a simpler method than conventional purse string suture. In our experience, tranumbilical multi-port laparoscopic orchiopexy with “8” pattern suture fixed bilateral patent processus vaginalis is relatively easy to perform with smaller scar and less pain. We propose the novel ligation as a more viable treatment option for pediatric cryptorchidism with bilateral patent processus vaginalis.

## Figures and Tables

**Table 1:** Character of two groups.

	LESS group	con Group	P value
**Mean age(range) year**	1.52±0.63(1~3)	1.62±0.74(1~3)	0.47
**Left/Right**	17/25	25/34	0.5639
**Cryptorchid abdominal Testis/ Cryptorchidism of inguinal canal**	3/39	5/54	0.558

**Table 2:** Summary of intraoperative and postoperative data of LESS versus laparoscopic orchiopexy.

	Operation time (min)	hospitalization days	Pain scores (6 hours after operation)	Pain scores (1day)	Pain scores (2day)	Pain scores (3day)	POSAS scores at 6 months
**LESS(n=42)**	35.85±4.02	4.40±1.39	2.54±0.56	2.40±1.04	1.60±0.73	0.81±0.59	6.83±0.82
**Con (n=59)**	36.01±4.08	4.47±1.31	2.69±1.07	2.63±0.93	2.02±0.86	1.98±0.92	7.64±1.09
**T**	-0.195	-0.256	-0.737	-1.13	-2.57	-7.26	-4.05
**P**	0.846	0.799	0.463	0.261	0.11	0.00	0.00

POSAS: Patient and Observer Scar Assessment Scale.
